# Understanding Engagement With Incident Reporting Systems Among NHS Healthcare Professionals: A Cross-Sectional Study

**DOI:** 10.7759/cureus.97060

**Published:** 2025-11-17

**Authors:** Uday Mahajan, Salman Shoukat Ali Parpia, Meraj Akhtar, Ria Gupta, Vibhore Gupta

**Affiliations:** 1 Trauma and Orthopaedics, Queen Elizabeth Hospital Birmingham, Birmingham, GBR; 2 Accident and Emergency Medicine, Queen Elizabeth Hospital Birmingham, Birmingham, GBR; 3 Trauma and Orthopaedics, Nottingham University Hospitals National Health Service (NHS) Trust, Lincoln, GBR; 4 Emergency Medicine, University of Birmingham, Birmingham, GBR; 5 Emergency Medicine, Queen Elizabeth Hospital Birmingham, Birmingham, GBR

**Keywords:** clinical governance, feedback gap, incident reporting, nhs guidelines, patient’s safety

## Abstract

Background

Incident reporting systems such as Datix (London, UK) and Radar (Leeds, UK) are key components of the NHS's patient safety infrastructure. Whilst widely implemented, engagement with these systems varies across professional roles and settings.

Aim

The aim of the study is to explore how NHS healthcare professionals engage with incident reporting systems, identify perceived barriers, and gather suggestions for improvement.

Methods

A cross-sectional, anonymised online survey was distributed to doctors, nurses, allied health professionals, and support staff working in a single large NHS hospital located in an urban area in the UK. The questionnaire included both closed- and open-ended questions covering system familiarity, usage, barriers, and perceptions. Quantitative data were analysed descriptively, and qualitative responses were reviewed thematically.

Results

Seventy-three professionals responded, with doctors forming the majority. Most participants were familiar with incident reporting systems, but submission rates were low, and many had never received formal training. Feedback following reports was inconsistent, and many respondents were unsure whether their reports led to meaningful change. Whilst most viewed the system as a safety tool, a notable proportion associated it with complaints or blame. Participants suggested that improved training, simplified reporting processes, and regular feedback would enhance engagement.

Conclusion

Incident reporting systems remain underutilised by NHS staff due to both practical and cultural barriers. Addressing these through targeted training, streamlined processes, and visible organisational learning may help shift perceptions and improve reporting behaviour.

## Introduction

Incident reporting systems are central to patient safety efforts within the NHS [1]. They are designed to capture adverse events, near misses, and other concerns, enabling organisations to learn from incidents, identify patterns, and improve care [2]. However, despite their widespread availability, engagement with these systems remains inconsistent [3].

Previous research suggests that doctors are less likely to report incidents than nurses or allied health professionals (AHPs) [4,5]. This disparity may reflect differences in training, workplace culture, time pressures, or uncertainty about what qualifies as a reportable event. Moreover, the perception that incident reporting is associated with blame or disciplinary action may further discourage use-particularly among junior staff or internationally trained clinicians [6].

Understanding how healthcare professionals use and perceive incident reporting systems is essential to improving their effectiveness [7]. High-quality reporting depends not only on access to the system but also on user trust, feedback mechanisms, and a culture that supports learning rather than blame [8].

This study aimed to explore the experiences and attitudes of healthcare professionals working in the NHS towards incident reporting. By examining usage patterns, barriers, and suggestions for improvement, we hope to identify practical and cultural factors that influence reporting behaviour and inform strategies to promote safer and more responsive care.

## Materials and methods

Study design

This was a cross-sectional, anonymised online survey conducted to evaluate healthcare professionals' familiarity with, use of, and perceptions regarding NHS incident reporting systems. The study aimed to identify barriers to reporting, perceived utility of reporting systems, and staff suggestions for improvement.

Study population and sample size

Eligible participants included healthcare professionals working within a single large NHS hospital located in an urban area in the UK. This encompassed doctors (at all levels), nurses, AHPs, and healthcare support workers. The survey was open to all roles and specialties. Convenience sampling was used, and a total of 73 responses were received over the data collection period. Whilst no formal sample size calculation was performed, this number provided a diverse mix of roles and experience levels suitable for exploratory analysis.

Study measures

The survey instrument consisted of 22 questions and was designed using Microsoft Forms (Microsoft Corp., Redmond, WA, US) (Appendices). These included multiple-choice questions, Likert-scale ratings, and free-text responses. It captured information across four key domains. First, demographic and background data were collected, including participants’ role, specialty, years of experience working in the NHS, and country of primary medical qualification. Second, respondents were asked about their familiarity with and usage of incident reporting systems-how often they had submitted reports, the types of incidents reported, and whether they had received any formal training. Third, the survey explored perceptions of reporting systems, including views on their purpose, perceived effectiveness, and whether they were seen as safety tools or instruments of blame. Finally, participants were asked to identify barriers they faced when attempting to report incidents, how long reports typically took to complete, whether they received feedback, and what changes they believed would improve system use and engagement.

A pilot was conducted with six clinicians (doctors and nurses) to assess clarity and relevance. Feedback led to minor refinements in language and structure.

Survey distribution

The survey link was shared via NHS trust internal communications, clinical governance forums, and WhatsApp groups (Meta Platforms, Inc., Menlo Park, CA, US). Participation was voluntary and anonymous. No incentives were offered. Data collection ran over four weeks in September 2025.

Ethics statement

As the study did not involve patients, use identifiable personal data, or affect clinical care, it was deemed a service evaluation project under NHS Health Research Authority guidance. According to local trust policy, formal ethical review was not required. Participation was voluntary, and submission of the form was taken as implied consent.

Statistical analysis

Quantitative data were exported to Microsoft Excel (Microsoft Corp.) and analysed using descriptive statistics. Frequencies and percentages were calculated for all categorical variables, including professional role, training background, and experience level. Due to the relatively small and unbalanced sample sizes across subgroups, no formal statistical tests (e.g., chi square or Fisher’s exact test) were performed to compare differences between groups. Instead, notable trends and contrasts were described narratively. Qualitative free-text responses were analysed thematically. Two independent reviewers coded the responses, identified common themes, and resolved any disagreements through discussion to ensure consistency and rigour.

## Results

Seventy-three healthcare professionals responded to the survey, with the majority being doctors, followed by nurses and a smaller number of AHPs and support workers. Whilst a full breakdown is shown in Table 1, over half of the participants had more than three years of NHS experience, and the majority obtained their primary qualification outside the UK. The emergency department was the most common work setting among respondents.

**Table 1:** Demographics of the participants AHPs: allied health professionals; HCSWs: healthcare support workers

Variable	n (%)
Total respondents	73 (100%)
Doctors	44 (60%)
Nurses	20 (27%)
AHPs	4 (5%)
HCSWs	3 (4%)
UK-trained	27 (37%)
EU-trained	5 (7%)
Non-EU-trained	41 (56%)
NHS experience < 1 year	7 (10%)
1–3 years	27 (37%)
4–7 years	17 (23%)
>7 years	22 (30%)

Most participants described themselves as familiar with incident reporting systems such as Datix (London, UK) or Radar (Leeds, UK), and two-thirds had submitted at least one report in the past year. However, submission frequency was typically low, with many reporting only a single incident. Whilst awareness was relatively high, confidence and engagement were more limited, suggesting that familiarity does not necessarily translate into routine use.

Patient safety concerns dominated the types of incidents reported, although delays in care, staff professionalism, and equipment failures also featured frequently (Table 2). This reflects the wide scope of what clinicians consider worthy of reporting, though there was considerable variability in understanding of what qualifies as a reportable event.

**Table 2:** Reporting experience

Metric	n
Submitted a report in the past 12 months	50
Reports submitted: 1	29
Reports submitted: 2–5	15
Reports submitted: 6–10	5
Reports submitted: >10	12
Familiar with the reporting system	68 (93%)
Received training at induction	12
Never received any training	29
Time to complete report: 5–20 min	45

Formal training appeared inconsistent. Only a small proportion of respondents reported receiving structured education during induction, whilst many had never received any training at all. Time required to submit reports was also a common concern, with most reporting that it took at least 10 minutes or more. Given the workload pressures in clinical settings, this likely contributes to underreporting.

Interestingly, although most participants believed the primary purpose of incident reporting was for learning and improvement, a significant minority perceived it as punitive or bureaucratic. This mismatch between the intended and perceived function of the system may hinder open engagement. The majority viewed it as a safety tool, but more than one in four associated it with complaints rather than learning.

Feedback after submission was highly variable. Only a fifth of participants reported consistently receiving feedback, and many had never received any. Where feedback was received, it was often seen as only ‘somewhat useful’, and many doubted whether reports led to meaningful change. This lack of visibility around outcomes may reduce motivation to report, especially for frontline staff.

**Figure 1:**
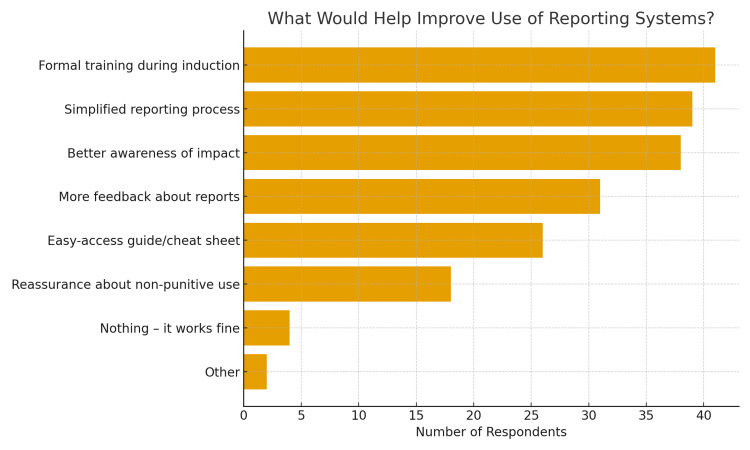
What participants said would help improve the use of incident reporting systems

Reasons for not reporting included uncertainty about what to report, lack of time, and fear of repercussions. Even among those who had submitted reports, many highlighted frustration with the system’s complexity or perceived lack of action. Respondents suggested improvements such as better induction training, simpler systems, and more consistent feedback (Figure 1). The average rating for system usability was only 5.1 out of 10, highlighting general dissatisfaction.

Overall, the findings suggest that whilst incident reporting systems are widely available and theoretically supported, their impact is limited by a combination of practical and cultural barriers. Improving training, usability, and feedback loops may enhance engagement and help shift perceptions from blame to learning.

## Discussion

This survey highlights ongoing challenges in how healthcare professionals engage with incident reporting systems in the NHS. Whilst the majority of respondents were familiar with systems, routine use remains limited and perceptions vary widely depending on professional role, training background, and individual experience.

Doctors, particularly those trained outside the UK, appeared less engaged with reporting systems than nurses or AHPs. This echoes findings from previous studies suggesting that awareness does not necessarily translate into action [4]. A major contributor may be the lack of structured, role-specific induction or follow-up support. Less than one in five respondents had received formal training, despite most acknowledging the importance of incident reporting for safety and learning. For internationally trained doctors, additional barriers may include unfamiliarity with NHS systems, limited exposure to incident reporting in prior settings, and language or communication challenges. Some may also fear professional or regulatory consequences, particularly those on visas or early in their General Medical Council (GMC) registration process [9]. These factors may contribute to reluctance to engage, highlighting the need for tailored support and clear reassurances around the non-punitive intent of reporting systems.

Although patient safety incidents were the most commonly reported type, participants also raised a broad range of concerns-from delays in care to professionalism issues-suggesting an awareness of the wider scope of safety culture. Yet, the overall number of reports per respondent remained low, and many cited uncertainty about what to report or how to do it. These are actionable gaps that could be addressed with targeted education. For instance, enhancing clinicians’ understanding of reporting criteria and processes could significantly improve both the quantity and quality of submissions [10,11].

Although patient safety incidents were the most commonly reported type, a range of concerns-from delays in care to professionalism-were also raised, reflecting broad awareness of safety culture. Despite this, reporting levels remained low, often due to uncertainty about what or how to report. Whilst targeted training could address knowledge gaps [9,10], broader interventions are also needed. Promoting a just culture, visible leadership support, and mentorship-particularly for internationally trained staff-may help foster a more open and confident reporting environment.

Importantly, the perceived purpose of these systems varied. Whilst most participants viewed reporting as a tool for learning, a significant proportion saw it as complaint-focused or punitive. This perception gap may be a key barrier to open and honest reporting. True cultural change requires that healthcare workers not only be told these systems are supportive but also see that reflected in feedback and outcomes. A lack of consistent and meaningful feedback following incident submissions has been identified as a critical factor undermining staff motivation and trust in the reporting process [12,13].

Feedback remains a major weakness. Many respondents either never received follow-up or found the feedback unhelpful. This aligns with a recurring message: that without visible results or acknowledgment, reporting feels futile. As reflected in the free-text comments, many clinicians want to feel their reports are heard and lead to action. This reinforces the understanding that for incident reporting to be effective, a transparent and responsive feedback loop is essential, demonstrating that reported issues contribute to systemic improvements rather than merely disappearing into a bureaucratic void [14,15]. In most cases, feedback is provided by the governance lead or the staff member responsible for managing the incident. However, local clinical leaders-such as ward managers, department heads, or educational supervisors-can play a key role in reinforcing and contextualising that feedback. Engaging them through clearer ownership, regular governance discussions, and making feedback part of routine team meetings may help close the loop more effectively and strengthen staff trust in the reporting process.

The most popular suggestions for improving the system were simple and practical: better training during induction, a clearer and quicker online interface, and more consistent feedback. A visual summary of these preferences (Figure 1) underscores the fact that many barriers are modifiable. Improving user experience and communication could significantly enhance engagement without the need for a complete system overhaul. Addressing the observed deficiencies, particularly the inadequate feedback mechanisms and the perceived futility of reporting, is crucial for fostering a culture where incident reporting is seen as a valuable learning opportunity rather than a punitive exercise [13,16].

Ultimately, this survey suggests that practical improvements-such as easier forms and faster processes-must go hand-in-hand with cultural shifts that promote trust, psychological safety, and visible learning from incidents. Encouragingly, most participants still believe in the value of incident reporting. The challenge now is making that belief easier to act on. This involves moving beyond merely collecting data to actively utilizing incident reports for systemic improvement and demonstrating tangible outcomes, as called for by both managers and incident reporting coordinators [13].

This study has some limitations. The sample size was modest and drawn from a single large urban NHS hospital, which may limit generalisability, as reporting culture can vary between organisations and regions. Participation was voluntary, which may have introduced selection bias, and the use of self-reported data carries the risk of recall or response bias. Despite these limitations, the inclusion of a balanced mix of doctors, nurses, and AHPs, as well as participants trained both within and outside the UK, strengthens the exploratory value and representativeness of the findings.

## Conclusions

This study provides insight into how healthcare professionals across different roles engage with NHS incident reporting systems such as Datix and Radar. Whilst most respondents recognised their value for learning and patient safety, actual usage was limited and shaped by a combination of practical barriers and cultural perceptions. Lack of training, inconsistent feedback, and the time burden of reporting were key obstacles, alongside concerns about whether reports lead to meaningful change. Perceptions of the system as punitive rather than constructive remain a significant challenge, particularly among doctors and internationally trained staff.

Encouragingly, participants offered practical, achievable suggestions-many of which centred on better induction, improved usability, and feedback that closes the learning loop. Addressing these areas may improve engagement, rebuild trust, and ensure that incident reporting fulfils its purpose as a core tool for quality improvement and safer care.

## Appendices

Survey: Use of Incident Reporting Systems (e.g., Datix/Radar) Among Medical Professionals

This short anonymous survey aims to explore how medical professionals engage with NHS incident reporting systems such as Datix or Radar. Your input will help identify trends, barriers, and areas for improvement in training and awareness.

1. Demographics (for analysis purposes only)

• Professional role:

  - Doctor

  - Nurse

  - Allied Health Professional (e.g., physiotherapist and OT)

  - Healthcare Support Worker

  - Other: ___________

• Job title/grade (e.g., FY1, SHO, Registrar, and Consultant):

• Specialty:

• Years of experience working in the NHS:

  - <1 year

  - 1-3 years

  - 4-7 years

  - >7 years

• Where did you complete your primary medical qualification?

  - UK

  - EU (outside UK)

  - Non-EU (international graduate)

2. Experience with incident reporting systems

• How familiar are you with systems like Datix or Radar?

  - Very familiar

  - Somewhat familiar

  - Not familiar at all

• Have you ever submitted an incident report?

  - Yes

  - No

• (If yes) Roughly how many incident reports have you submitted in the past 12 months?

  - 1

  - 2-5

  - 6-10

  - >10

• What kind of incidents have you reported? (Tick all that apply)

  - Patient safety incident

  - Staff behaviour/professionalism

  - Equipment failure

  - Medication error

  - Documentation issue

  - Delay in care

  - Other: ___________

• Have you received formal training in using Datix/Radar or any other incident reporting system?

  - Yes - as part of induction

  - Yes - in a separate session

  - No - I learned informally

  - No - I have never been trained

3. Perception of reporting systems

• What do you believe is the primary purpose of these systems? (Tick all that apply)

  - Blame or disciplinary action

  - Learning and service improvement

  - Tracking clinical trends

  - Mandatory documentation

  - Protection against litigation

  - Unsure

• Do you view incident reporting systems primarily as:

  - Safety improvement tools

  - Complaint tools

  - Legal protection

  - Administrative burden

  - Not sure

• Do you receive feedback or follow-up after submitting a report?

  - Always

  - Sometimes

  - Rarely

  - Never

• Do you believe incident reporting is more routinely used by:

  - Nursing staff

  - Medical staff

  - Equally by both

  - Not sure

4. Barriers and suggestions

• If you’ve never submitted a report, why not? (Tick all that apply)

  - I haven’t encountered a reportable incident

  - I don’t know how to submit one

  - I don’t have time

  - I’m unsure what counts as a reportable incident

  - Fear of repercussions

  - Lack of feedback makes it feel pointless

  - Language or system unfamiliarity

  - Other: ___________

• What would help you use these systems more effectively? (Tick all that apply)

  - Formal training during induction

  - Easy-access guide or cheat sheet

  - Reassurance about non-punitive use

  - More feedback about submitted reports

  - Better awareness of its impact

  - Nothing-it works fine as it is

  - Other: ___________

5. Open feedback (optional)

• In your own words, how could the use of incident reporting systems be improved at your workplace?

  ____________________________________________________________________________

  ____________________________________________________________________________

  ____________________________________________________________________________
